# Environmental variables and definitive host distribution: a habitat suitability modelling for endohelminth parasites in the marine realm

**DOI:** 10.1038/srep30246

**Published:** 2016-08-10

**Authors:** Thomas Kuhn, Sarah Cunze, Judith Kochmann, Sven Klimpel

**Affiliations:** 1 Goethe-University, Institute for Ecology, Evolution and Diversity; Senckenberg Biodiversity and Climate Research Centre; Senckenberg Gesellschaft für Naturforschung; Max-von-Laue-Str. 13, D-60438 Frankfurt/Main, Germany

## Abstract

Marine nematodes of the genus *Anisakis* are common parasites of a wide range of aquatic organisms. Public interest is primarily based on their importance as zoonotic agents of the human Anisakiasis, a severe infection of the gastro-intestinal tract as result of consuming live larvae in insufficiently cooked fish dishes. The diverse nature of external impacts unequally influencing larval and adult stages of marine endohelminth parasites requires the consideration of both abiotic and biotic factors. Whereas abiotic factors are generally more relevant for early life stages and might also be linked to intermediate hosts, definitive hosts are indispensable for a parasite’s reproduction. In order to better understand the uneven occurrence of parasites in fish species, we here use the maximum entropy approach (Maxent) to model the habitat suitability for nine *Anisakis* species accounting for abiotic parameters as well as biotic data (definitive hosts). The modelled habitat suitability reflects the observed distribution quite well for all *Anisakis* species, however, in some cases, habitat suitability exceeded the known geographical distribution, suggesting a wider distribution than presently recorded. We suggest that integrative modelling combining abiotic and biotic parameters is a valid approach for habitat suitability assessments of *Anisakis*, and potentially other marine parasite species.

The distribution of marine endohelminth parasites is influenced by a wide range of abiotic and biotic factors. While the development and dispersal of excreted propagules (eggs) is predominantly influenced by physical parameters (e.g. salinity^1^,^2^, ocean currents^3^, temperature^4^,^5^), distribution of endoparasitic intermediate and adult stages is largely shaped by the transmission pathways, which closely follow trophic interrelations between the parasites´ definitive, intermediate and transport hosts and their respective migrating behaviour^6^,^7^,^8^,^9^.

*Anisakis* is a genus comprising species of marine, zoonotic, endohelminth nematodes that gained first attention of researchers and the general public in the mid of the 20^th^ century, when Van Thiel *et al*.^10^ reported *Anisakis* parasites from a patient suffering from acute abdominal syndromes, a clinical picture later described as Anisakiasis^11^. The life cycle of the “whale worm” was recognized and broadly described only in the last 50 years; it is stated as heteroxenous (including more than one host) in marine habitats^12^,^13^,^14^,^15^. Adult *Anisakis* parasitize the digestive tract of toothed and baleen whales (Cetacea: e.g. Delphinidae, Ziphiidae, Physeteridae und Kogiidae)^9^,^16^,^17^ (Table 1). Typical intermediate hosts are invertebrates (e.g. calanoid Copepoda, Euphausiacea), which transmit the infective larvae along the food chain onto paratenic intermediate hosts such as cephalopods, teleost fishes and larger predators^13^,^18^,^19^,^20^,^21^,^22^.

Owing to the routine application of molecular techniques as diagnostic tools in biodiversity research, it is now accepted that the genus *Anisakis* contains nine distinct species, each with different ecological characteristics (e.g. host specificity) and human zoonotic hazardous potential^9^,^23^,^24^,^25^,^26^. The results of various studies indicate that there is a direct relationship between the prevalence and abundance of anisakid nematodes in their (paratenic) intermediate hosts and the occurrence and population size of their vertebrate definitive hosts (see McClelland^27^ and references therein) suggesting their host specificity to be the main external ecological attribute that determines both their range size and local abundance, and thus, influencing the epidemiology of human anisakiasis infections^27^,^28^,^29^.

Kuhn *et al*.^6^ presented the first modelling approach of the zoogeographical distribution of *Anisakis* spp. based on molecularly identified presence data, which has previously been displayed only on the basis of biogeographical occurrence data in form of so called “dot maps” (see review Mattiucci and Nascetti^9^, and references therein). This first modelling approach combined different aspects and principles of modelling techniques like alpha-hull and conditional triangulation which have been proven useful in the assessment of the conservation status of species before^30^,^31^. The authors strongly suggested to carefully re-evaluate the likelihood of anisakid infections in a given area due to the existence of species specific distribution patterns among the species of the genus *Anisakis*^6^. However, due to the lack of adequate data for marine environmental parameters and the relatively limited amount of molecularly documented presence data available at the time, the initial modelling approach only represented a rough estimate of the *Anisakis* species’ ranges.

With GMED (The Global Marine Environment Datasets), available since 2014, standardized data of environmental variables are now available, thus, offering an opportunity to model the geographical distribution of marine species^32^. GMED provides climatic, biological and geophysical environmental layers of present day, past and future environmental conditions from different sources in a standardized format, resolution and extent.

The first aim of the present study was to re-model the distribution of *Anisakis* using updated molecular occurrence data extracted from the scientific literature and a state-of-the-art habitat suitability modelling approach based on environmental parameters (abiotic factors) available to date (Table 2). Furthermore, information on the potential distribution of definitive host species (biotic factors, Table 3) was incorporated in the habitat suitability modelling in order to further refine habitat suitability assessment.

## Results

### Habitat suitability modelling

**Figure 1:**
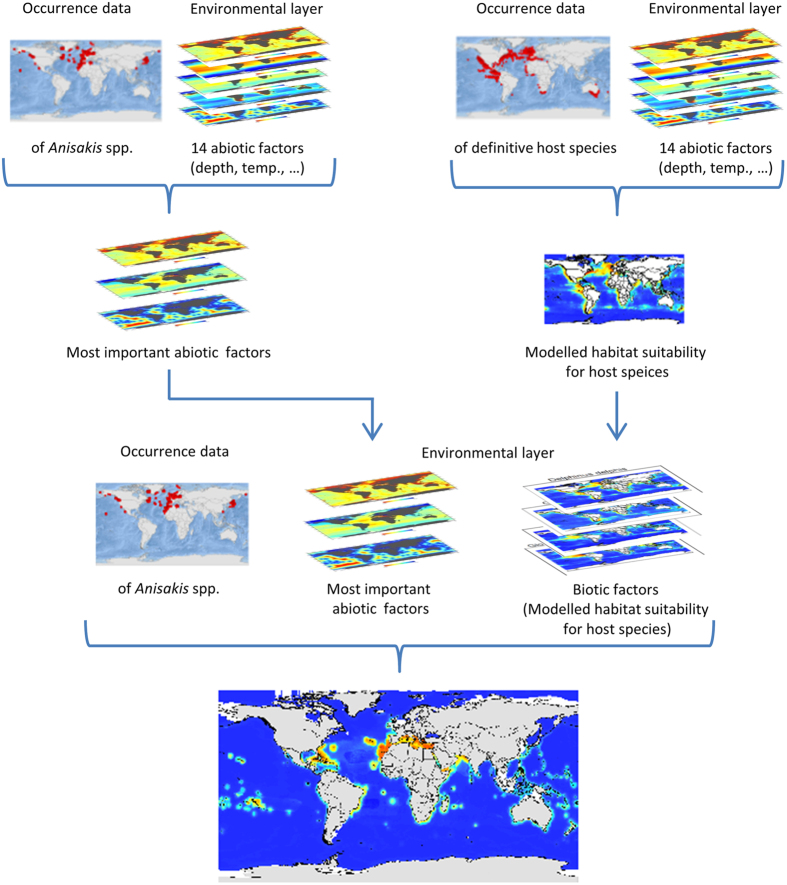
Chart of the modelling approach. In intermediate step 1 (IS1), the habitat suitability for the *Anisakis* species were modelled depending on the occurrence record of the respective species and 14 marine environmental variables (Table 2) in order to find the most important abiotic factors. In intermediate step 2 (IS2) the habitat suitability for the definitve host species of the *Anisakis* species were modelled based on the occurrence record provided by Aquamaps^35^ and the same 14 marine environmental variables (Table 2). The habitat suitability for the nine *Anisakis* species was finally modelled based on both: abiotic factors (land distance, depth, salinity, sea surface temperature (mean, range), primary production) and biotic variables (i.e. the modelled habitat suitability of the respective definitive host species). For visualization, maps were built using Esri ArcGIS 10.3 (www.esri.com/software/arcgis).

Based on the results of the Maxent permutation importance, as a measure for variables’ contribution to the habitat suitability model, the following six variables were identified as most important abiotic factors for the potential distribution of *Anisakis* species: land distance, mean sea surface temperature, depth, salinity, sea surface temperature range as well as primary production. These six variables were used as abiotic factors together with the modelled habitat suitability for the definitive host species of the respective *Anisakis* species as biotic factors to model the habitat suitability for the *Anisakis* species in the final model (Fig. 1,[Fig f2], Table 2).

The modelling results for the nine *Anisakis* species are displayed in Figs 3, 4, 5. Warmer colours indicate a higher modelled habitat suitability and thus, a higher occurrence probability for the considered *Anisakis* species. Additionally, the occurrence data for the *Anisakis* species (Figs 3, 4, 5: dot maps in column 1) and the definitive host species diversity (Figs 3, 4, 5: column 2) for the respective *Anisakis* species are visualized. For the definitive host species diversity, we summed up the binary modelling results (according to the sensitivity equals specificity optimization criterion)^33^ for all recorded definitive host species of the respective *Anisakis* species. A high definitive host diversity means that several of the recorded definitive host species are modelled to find suitable habitat conditions in a certain region.

“Area under the Receiver Operator Curve (AUC)” values for the Maxent models of the nine *Anisakis* species are summarized in Table 3. The AUC value is a commonly used measure of model performance independent of a threshold. The AUC values scores between 0 and 1. Assuming unbiased data, higher values indicate that areas with high modelled habitat suitability tend to be areas of known presence and areas with lower modelled habitat suitability tend to be areas where the species is not known to be present^34^. An SDM with an AUC value of about 0.5 is assumed to be as good as a random model. For all considered *Anisakis* species, the AUC values are high (above 0.95).

Table 3 lists the number of occurrence records for each *Anisakis* species as well as the number of environmental variables (6 abiotic variables and a varying number of hosts (biotic variable) depending on the species) used for the modelling. The habitat suitability for five definitive host species (i.e. *Feresa attenuata*, *Sotalia fluviatilis*, *S. guianensis*, *Mesoplodon bowdoni*, *M. europaeus*) could not be modelled as for these species insufficient (less than 10) or no occurrence records were available from AquaMaps^35^. Thus, for some *Anisakis* species the number of considered definitive host species variables is less than the number of recorded definitive host species (Table 1).

### Zoogeography of ***Anisakis*** spp

A comparison of the occurrence data dot maps (Figs 3, 4, 5: dot maps) and the modelled habitat suitability (Figs 3, 4, 5: third column) shows that the potential area of distribution (=high modelled habitat suitability) extends in some cases the recorded geographical area as documented by the occurrence data points. According to sampling data currently available, *Anisakis simplex* sensu strictu, for example, was exclusively found in fish hosts of the northern hemisphere, and here mainly along the North/North-East Atlantic as well as in waters of the West and East Pacific Ocean, whereas the habitat suitability maps suggest it to be present also in more southern areas, e.g. between the Antarctic Peninsula and South America and the waters around New Zealand.

The modelled host diversity patterns (Figs 3, 4, 5: column 2) differ to some extent from the modelled habitat suitability for the *Anisakis* species (Figs 3, 4, 5: column 3), i.e. the distribution of the parasites do not always overlap with the diversity hotspots of the definitive hosts and *vice versa*. This is particularly striking in the above mentioned area of the Central East Pacific (Figs 3, 4, 5). Hotspots with both high data density and diversity (all nematode species) can be found in the North/North-East Atlantic, the Mediterranean and the West and East Pacific Ocean along coasts of Japan/China and North America, respectively. Hotspots of cetacean definitive diversity (Figs 3, 4, 5: column 2) are located in the area of the tropics and subtropics, especially along the west coast of Central America (e.g. Mexico, Guatemala, Ecuador, Peru), Indian Ocean (Bay of Bengal, Indonesia, Thailand) and China, Japan and New Zealand. Areas with lower densities are located along the south-eastern tip of South America, the Antarctic Peninsula as well as other regions of the Southern Ocean.

### Correlation Analysis

**Figure 2 f2:**
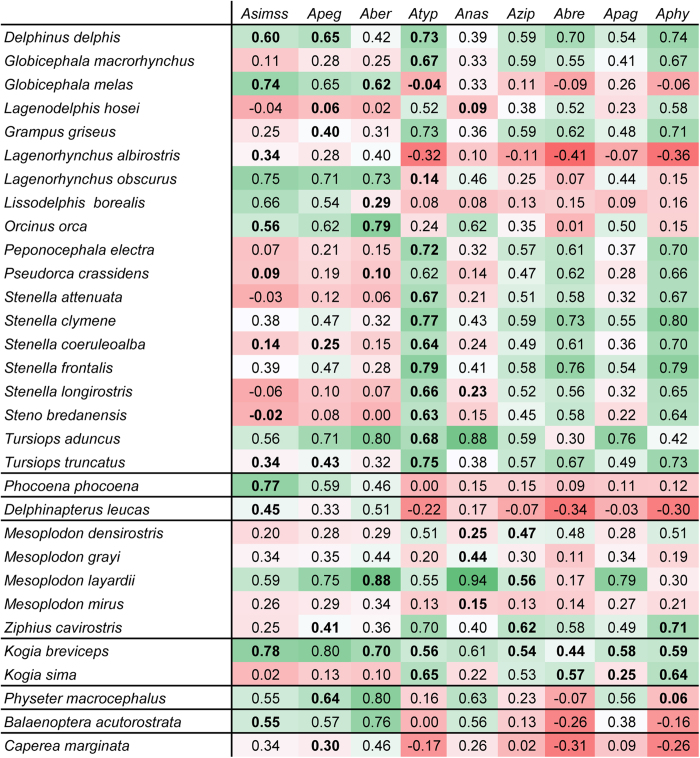
Parasite-host correlation analyses. Spearman correlation coefficients between the modelled habitat suitabilities of the nine *Anisakis* species based on 14 abiotic factors (IS1) and the modelled habitat suitability for the 37 definitive host species based on the same 14 abiotic factors (IS2). Bold correlation coefficients relate to *Anisakis*-definitive host species pairs that are known to interact with each other. Colour intensities indicate correlation strength: dark red: strong negative correlation, dark green: strong positive correlation *Asimss*: *A. simplex* s.s.; *Apeg*: *A. pegreffii*; *Aber*: *A. berlandi*; *Atyp*: *A. typica*; *Azip*: *A. ziphidarum*; *Anas*: *A. nascetti*; *Apag*: *A. paggiae*; *Abre*: *A. brevispiculata*; *Aphy*: *A. physeteris*.

Spearman correlation coefficients (r_s_) between the modelled habitat suitability of *Anisakis* species obtained by IS1 and definitive hosts obtained by IS2 were calculated for every combination of parasite and host (Fig. 2). Values varied between a minimum of r_s_ = −0.41 (*A.brevispiculata* – *Lagenorhynchus albirostris*) and a maximum of r_s_ = 0.94 (*A. nascettii* – *Mesoplodon layardii*). In some cases, positive correlations were obtained, even if no parasite-host-interaction between certain combinations has been documented from the literature so far (e.g. *A. pegreffii*/*Globicephala melas* [0.65], *A. paggiae*/*Tursiops truncatus* [0.79]). Simultaneously, not every documented parasite-host-combination, despite documented host record, yielded positive correlation values (e.g. *A. simplex s.s./Steno bredanensis* [−0.02], *A. typica*/*Globicephala melas* [−0.04]).

## Discussion

### Current distribution and habitat suitability modelling of Anisakis spp

The genus *Anisakis* comprises two major clades; the first clade includes the *A. simplex*-complex (*A. simplex* sensu stricto [s.s.], *A. pegreffii*, *A. berlandi*) as well as *A. typica* and two sister-species *A. nascettii* and *A. ziphidarum*^23^. The second clade consists exclusively of the *A. physeteris*- complex (*A. brevispiculata*, *A paggiae*, *A. physeteris*)^6^,^23^. Both *A. simplex*-complex and *A. physeteris*-complex are considered cryptic species, distinguishable only by means of molecular analyses as well as slight morphological differences (e.g. tail length/total body length ratio; spicule length)^23^.

The distribution (dot maps) of *Anisakis* species generated in this study coincides in large parts with results of earlier studies^6^,^9^ suggesting an uneven occurrence of the nine species among the different climate zones and oceans. Some new records were added, however, those are mostly located in already known endemic areas.

Hot spots of occurrence records were found in the Mediterranean region, in the region of Japan, along the North-American coasts as well as in the waters of the North-Atlantic; areas with extensive fisheries and economically (and consequently scientifically) important fish species. Hot spots of host diversity are not inevitably congruent with these areas, mostly because reliable occurrence data for the definitive hosts usually stem from regions with a good accessibility and a scientific and public interest (e.g. “whale watching”, scientific research cruises, cruise itineraries), which do not necessarily overlap with the areas where intermediate fish hosts are caught.

**Figure 3:**
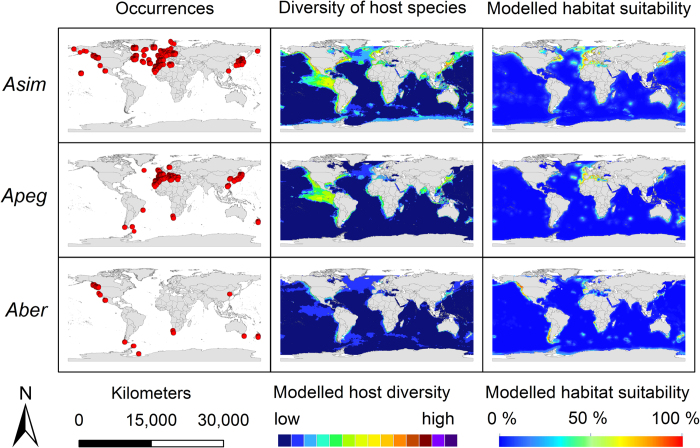
Modelling results. First column: *Anisakis* occurrence records used for modelling displayed as dot maps; second column: diversity of definitive host species of the respective *Anisakis* spp. as a sum map of the dichotomous modelling results for the SDM results for the definitive host species obtained by IS2; third column: modelled habitat suitability for the nine *Anisakis* spp. based on abiotic and biotic variables. *Asim*: *A. simplex* s.s.; *Apeg*: *A. pegreffii*; *Aber*: *A. berlandi*. For visualization, maps were built using Esri ArcGIS 10.3 (www.esri.com/software/arcgis).

The close phylogenetic relationship between some of the species is mirrored in similar modelling results using biotic and abiotic variables. *Anisakis simplex* s.s. has exclusively been recorded by means of molecular methods from hosts in the northern hemisphere, mainly in the Atlantic and Pacific Ocean as well as in the Western Mediterranean (Fig. 3). The distribution of data points indicates a main distribution area of this nematode species in the entire North Atlantic. Below 20°N latitude, no evidence of the species’ occurrence has been found to date. The map of the habitat suitability shows some areas of the southern hemisphere (e.g. New Zealand), where occurrences would be expected due to suitable local environmental conditions. An accumulation of occurrences along Japanese coastal waters, which was also observed for *A. pegreffii* (see below), can most likely be explained by increased economic research interests in potential harmful organisms in commercially highly significant and often consumed raw fish species.

*Anisakis pegreffi* shows a disjunct distribution, with a lack of evidence along the North American West Coast and some additional findings between South America and the Antarctic Peninsula and South Africa and New Zealand.

The distribution of *A. berlandi*, along the Pacific Northwest Coast, the Southern Ocean, the Weddell Sea and the coast of South Africa is comparable to that of the two closely related sister species, *A. simplex* s.s. and *A. pegreffii*. The close phylogenetic relationship of these three species is reflected in a similar distribution pattern (Fig. 3). Small differences between the modelling results for these three sister species probably arise due to different species specific host preferences (see Tables 1 and Fig. 2).

**Figure 4:**
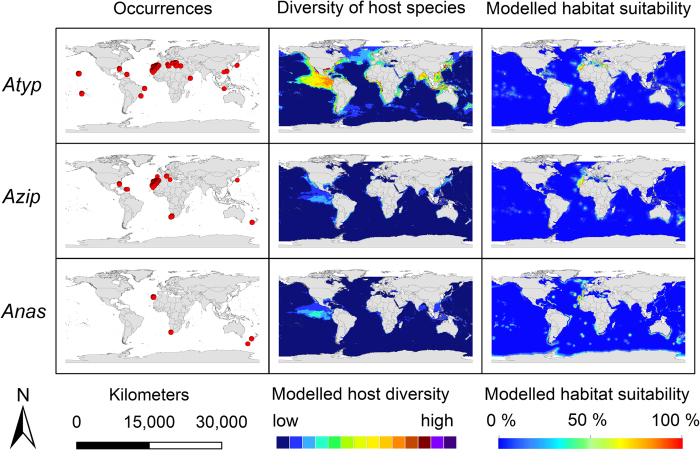
Modelling results. First column: *Anisakis* occurrence records used for modelling displayed as dot maps; second column: diversity of definitive host species of the respective *Anisakis* spp. as a sum map of the dichotomous modelling results for the SDM results for the definitive host species obtained by IS2; third column: modelled habitat suitability for the nine *Anisakis* spp. based on abiotic and biotic variables. *Atyp*: *A. typica*; *Azip*: *A. ziphidarum*; *Anas*: *A. nascetti*;. For visualization, maps were built using Esri ArcGIS 10.3 (www.esri.com/software/arcgis).

Due to its characteristic distributional pattern along the tropics and subtropics, *A. typica* stands out from other members of the genus (Fig. 4). The model proposes a circumglobal distribution with temperature and land distance constituting decisive factors. The highest correlations between definitive host and parasite distribution arise for the warm-water adapted species of the genus *Stenella* as well as coast-associated Delphinids such as *Tursiops truncatus* and T. *aduncus*, suggesting their large influence on the distribution of *A. typica*.

*Anisakis ziphidarum* and the sister species *A. nascettii* were sporadically detected in the Central and South-East Atlantic as well as in New Zealand and the Mediterranean Sea (Fig. 4). *A. ziphidarum* was further detected in Japanese waters. *Anisakis nascettii* was described only very recently by Mattiucci, Paoletti et Webb (2009)^36^, which explains the low number of molecular evidence. For both species, potential habitat suitability was modelled at few locations off their known distribution areas. In comparison with other members of the genus, both species are considered to be host specific with only a small number of definitive host species documented.

**Figure 5:**
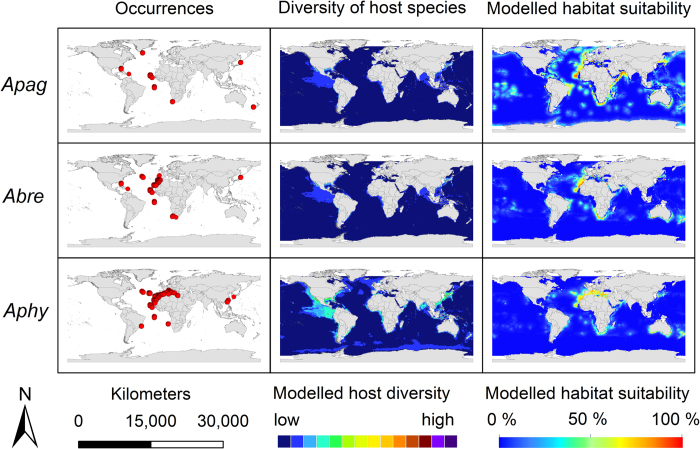
Modelling results. First column: *Anisakis* occurrence records used for modelling displayed as dot maps; second column: diversity of definitive host species of the respective *Anisakis* spp. as a sum map of the dichotomous modelling results for the SDM results for the definitive host species obtained by IS2; third column: modelled habitat suitability for the nine *Anisakis* spp. based on abiotic and biotic variables. *Apag*: *A. paggiae*; *Abre*: *A. brevispiculata*; *Aphy*: *A. physeteris*. For visualization, maps were built using Esri ArcGIS 10.3 (www.esri.com/software/arcgis).

The relatively homogeneous appearance of the three representatives of the *Anisakis physeteris*-complex (*A. physeteris*, *A. brevispiculata*, *A. paggiae*) in the tropical and subtropical Atlantic, and occasionally in Japan/China and New Zealand (only *A. paggiae*), reflects their close phylogenetic relationship^9^,^37^ (Fig. 5). They show, similar to *A. ziphidarum* and *A. nascettii*, increased host specificity; based on literature clear host preferences occur for kogiid *Kogia breviceps* and *K. sima* while *A. physeteris* is the only type of the complex which additionally parasitizes the sperm whale *Physeter macrocephalus*^9^. Within the verified host range, highest correlation is noted for *Kogia breviceps* (r_s_ = 0.44–0.59). Increased correlation values for definitive hosts which are not in the known host range of the three types (e.g. *Delphinus delphis*, *Tursiops truncatus*) (see Fig. 2) can represent a “lack-of-data” (i.e., occurrence of specific species has not been recorded so far), or point to a lack of host suitability for the parasite.

### Drivers influencing Anisakis spp. distribution

Land distance, mean sea surface temperature, depth, salinity, sea surface temperature range as well as primary production, were identified as most important abiotic variables (variable important analysis) impacting the distribution of *Anisakis*. Klimpel and Rückert^38^ demonstrated the influence of physical systems (mixed and stratified waters), and in particular hydrographic conditions like fronts, on the abundance and distribution of marine helminths of the raphidascarid nematode *Hysterothylacium aduncum* from the North Sea. Fueled by an increased primary and secondary production, permanent upwelling of nutrients resulted in an accumulation of predators and prey in the vicinity of halo/thermocline fronts, and thus, favouring the transmission of parasites among hosts. Højgaard^4^ found that hatching time of *Anisakis simplex* eggs varied inversely with temperature and that survival time increased with salinity but decreased with temperature, supporting the hypothesis that *Anisakis simplex* is rather adapted to (off-shore) pelagic marine environments with high salinity. Both temperature and salinity were identified crucial in the variable importance analyses of this study. The influence of land distance and depths is probably an effect of the individual association of certain definitive host groups to either offshore or inshore marine habitats. Sperm whales, for example, usually inhabit deep sea habitats and tend to migrate in off-shore waters over large distances resulting in the dispersal of parasite propagules (e.g. *A. physeteris*) in offshore pelagic waters^39^. In contrast, the delphinid species *Tursiops aduncus* and *T. truncatus*, common hosts of *A. typica* (Table 1 and Fig. 2), are rather coast-associated favouring the distribution of this parasite along in-shore habitats.

### Habitat suitability modelling approach

The modelled habitat suitability using abiotic environmental parameters and biotic host distribution data reflects the observed distribution pattern for all nine *Anisakis* species quite well. The generally high accordance between the distribution of occurrence data and modelling results is supported by high AUC values. As the AUC values depend on the number of considered occurrence records the AUC values may not be used in order to compare the performance of modelling results between species.

Sampling bias is one of the issues that has to be considered when modelling species’ distribution using correlative approaches and may have affected our data here, i.e. *Anisakis* and definitive host species occurrences. Occurrence data are always affected by a sampling bias, e.g. species are commonly recorded in specific areas more often than in others due to a larger interest in that region compared to others, resulting in unequal probabilities of records. Hot spots of recorded occurrence and hotspots of actual occurrences may thus differ, which may yield a false representation of the species’ niche reducing the reliability of the modelling results.

In some regions (e.g. South Atlantic for *A. simplex* s.s./*A. pegreffii*) the area with high modelled habitat suitability exceeds the area with recorded presences of the *Anisakis* species. This could have several causes: Despite suitable habitat conditions, *Anisakis* species do not occur in these regions which may be due to a potential dispersal limitation of the *Anisakis* species or the associated host species (e.g. migratory vs. more stationary cetacean definitive hosts). A mismatch could also be caused by limited sampling efforts, i.e., that the *Anisakis* species occur in these regions with high modelled habitat suitability but no (molecular, to cryptic species) occurrence has been recorded there to date (sampling bias). A third reason might be that a crucial factor relevant for *Anisakis* habitat requirements was not considered in the modelling. Overall, it is not clear which of these factors might best explain the “overprediction”.

The present study confirmed the postulated zoogeographical ranges of *Anisakis* spp in certain climate zones and oceans. The present approach using abiotic environmental parameters and biotic host distribution data reflects the observed distribution pattern for all nine *Anisakis* species quite well. Land distance, mean sea surface temperature, depth, salinity, sea surface temperature range and primary production were found to be the driving abiotic factors, which are in particular relevant for early marine parasitic life stages. However, final hosts are indispensable for a parasite’s reproduction in a certain area. Thus, the integration of host species distribution is considered crucial when modelling habitat suitability for parasites and abiotic and biotic factors should therefore be used in an integrated approach and are equally valid for risk assessments of zoonotic diseases.

## Methods

### Species distribution modelling approaches

Habitat suitability models, also called species distribution models (SDMs) or environmental niche models (ENMs), are correlative approaches that model the potential geographical range of a species subjected to various environmental variables^40^. Based on the information in which location a species is observed to be present or absent, and the environmental conditions prevailing there, the species-environment-relationship is estimated as a function of the relative habitat suitability in relation to the considered environmental conditions. The modelled species-environment-relationship (niche function) can then be projected onto maps showing the relative habitat suitability or occurrence probability for the considered species.

Here, the maximum entropy niche modelling approach (implemented in the freeware MAXENT)^41^,^42^ was used with the default settings but only using linear, quadratic and product features^43^. The MAXENT approach is one of the most commonly employed algorithms to model species potential ranges (e.g.^44^) and scores well in comparative studies (e.g.^45^). According to Baldwin^46^, the maximum entropy approach is relatively insensitive to spatial errors associated with location data, requires few locations to construct useful models, and performs better than other presence-only modelling algorithms. This may be in particular striking considering the fact that assessment of real absence data is not feasible for *Anisakis* species.

### Occurrence data

Habitat suitability modelling was carried out using only *Anisakis* occurrence data from studies that have performed molecular methods to guarantee unambiguous identification to (cryptic) species level (e.g. PCR-RFLP, SSCP, direct sequencing; MAE: Multilocus-Allozyme-Electrophoresis). In addition to the presence data that have been included in a former approach described in Kuhn *et al*.^6^, the ISI Web of Knowledge online database was searched according to a set of search terms (*Anisakis*, Anisakid) to assess the novel research articles in the field that have been published since 2011 and extract specific locality records. In total, 101 publications were considered in the present model^9^,^16^,^17^,^18^,^22^,^36^,^47^,^48^,^49^,^50^,^51^,^52^,^53^,^54^,^55^,^56^,^57^,^58^,^59^,^60^,^61^,^62^,^63^,^64^,^65^,^66^,^67^,^68^,^69^,^70^,^71^,^72^,^73^,^74^,^75^,^76^,^77^,^78^,^79^,^80^,^81^,^82^,^83^,^84^,^85^,^86^,^87^,^88^,^89^,^90^,^91^,^92^,^93^,^94^,^95^,^96^,^97^,^98^,^99^,^100^,^101^,^102^,^103^,^104^,^105^,^106^,^107^,^108^,^109^,^110^,^111^,^112^,^113^,^114^,^115^,^116^,^117^,^118^,^119^,^120^,^121^,^122^,^123^,^124^,^125^,^126^,^127^,^128^,^129^,^130^,^131^,^132^,^133^,^134^,^135^,^136^,^137^,^138^,^139^,^140^,^141^,^142^

37 cetacean species are currently recognized as definitive host for *Anisakis* (see Table 1). Occurrence records for these host species were derived from the online tool AquaMaps^35^ with a reduced spatial resolution of 1 decimal degree.

### Environmental data

Marine environmental data were obtained by the Global Marine Environment Datasets (GMED)^32^ (Table 2). It has been suggested that interpolation errors might arise in the North Polar regions, thus, cropped data sets with an extent up to 70°N were used.

The environmental data were loaded at a spatial resolution of 5 arc minutes (=0.083 decimal degree) and rescaled at a spatial resolution of 1 decimal degree computing the mean. This resolution is in accordance with the resolution of the occurrence records for the *Anisakis* species.

Of all marine environmental variables provided by GMED, only a subset of 14 environmental variables (Table 2) was considered for the further analysis. This choice of 14 abiotic predictor variables among all available marine environmental variables provided by GMED was based on correlation as well as ecological relevance. These 14 variables were used in an intermediate step (IS1), to model the habitat suitability of the nine *Anisakis* species with the primary aim to identify those variables that on average contributed most to a Maxent model. In order to identify the most important abiotic factors the Maxent models were run for each of the nine *Anisakis* species. The Maxent permutation importance (i.e. a measure for the variables’ contribution to the Maxent model) was calculated for each of the 14 abiotic factors and was transformed into ordinal rank scale for each of the nine *Anisakis* species. The six abiotic factors with the lowest median for all nine *Anisakis* species were taken as a final subset of abiotic factors in order to model the habitat suitability for the *Anisakis* species, together with the modelled habitat suitability of all known final host species as biotic factors (final model). This was done in order to reduce the number of predictor variables for the final modelling (integration of abiotic and biotic factors), thus, reducing the risk of overfitting. Based on the same subset of 14 abiotic factors (Table 2) as considered in IS1, the habitat suitability for each of the definitive host species (Table 1) was modelled in a second intermediate step (IS2). The habitat suitability for the nine *Anisakis* species was finally modelled based on the six abiotic factors found in the first intermediate step as well as on the modelled habitat suitability maps of all known final host species of the respective *Anisakis* species.

Here, definitive host distributions were included as anisakids are very specific to these, but less towards their intermediate hosts. “Finding” intermediate hosts refers to the mainly passive digestion by different crustaceans and fish, which are generally widely distributed in the oceans, thus, a much greater distribution could be expected and hence, area of habitat suitability leading to an overprediction of suited habitats for the different *Anisakis* species if based only on intermediate hosts.

In order to evaluate the modelling results, dot maps with the recorded occurrences of each of the nine *Anisakis* species were created. Additionally, host species diversity maps of the respective *Anisakis* species were built. For these maps, the continuous modelling results (from the intermediate step IS2) for the definitive host species were converted into binary data (1 – suitable habitat conditions, 0 – unsuitable habitat conditions) using the threshold that minimizes the difference between sensitivity and specificity^33^. These binary modelling results were then summed up for all known definitive host species of a certain *Anisakis* species resulting in definitive host diversity maps that show the number of definitive host species for the respective *Anisakis* species with modelled habitat suitability. For visualization, maps were built using Esri ArcGIS 10.3.

### Correlation analyses

Spearman correlation coefficients between the modelled habitat suitability for the different *Anisakis* species based on the 14 abiotic variables (IS1) and the modelled habitat suitability for the definitive host species based on the same 14 abiotic variables (IS2) were calculated to identify pairs of definitive host species and parasite species with a similar pattern of modelled habitat suitability. For pairs with a similar pattern of modelled habitat suitability a parasite-host interaction was assumed.

## Additional Information

**How to cite this article**: Kuhn, T. *et al*. Environmental variables and definitive host distribution: a habitat suitability modelling for endohelminth parasites in the marine realm. *Sci. Rep.* **6**, 30246; doi: 10.1038/srep30246 (2016).

## Figures and Tables

**Table 1:** Definitive hosts so far detected and molecularly verified for *Anisakis* spp.

Family	Definitive host	Parasite								
		*A. simplex*(s.s.)	*A. pegreffii*	*A. berlandi*	*A. typica*	*A. nascettii*	*A. ziphidarum*	*A. brevispiculata*	*A. paggiae*	*A. physeteris*
Delphinidae	*Delphinus delphis*	7	7		7					
	*Feresa attenuata*				7					
	*Globicephala macrorhynchus*				7					
	*Globicephala melas*	7		7	7					
	*Lagenodelphis hosei*		2			7				
	*Grampus griseus*		1							
	*Lagenorhynchus albirostris*	7								
	*Lagenorhynchus obscurus*				7					
	*Lissodelphis borealis*			7						
	*Orcinus orca*	7								
	*Peponocephala electra*				7					
	*Pseudorca crassidens*	7		7						
	*Stenella attenuata*				7					
	*Stenella clymene*				5					
	*Stenella coeruleoalba*	5	7		7					
	*Stenella frontalis*				7					
	*Stenella longirostris*				7	3				
	*Sotalia fluviatilis*				7					
	*Sotalia guianensis*				4					
	*Steno bredanensis*	2			7					
	*Tursiops truncatus*	1	7		7					
	*Tursiops aduncus*				6					
Phocoenidae	*Phocoena phocoena*	7								
Monodontidae	*Delphinapterus leucas*	7								
Pontoporiidae	*Pontoporia blainvillei*				7					
Ziphiidae	*Mesoplodon bowdoini*					8	8			
	*Mesoplodon densirostris*					3	7			
	*Mesoplodon europaeus*						7			
	*Mesoplodon grayi*					7				
	*Mesoplodon layardii*			7			7			
	*Mesoplodon mirus*					7				
	*Ziphius cavirostris*		7				7			1
Kogiidae	*Kogia breviceps*	2		9	5		9	7	7	7
	*Kogia sima*				10			3	7	2
Physeteridae	*Physeter macrocephalus*		7							7
Balaenopteridae	*Balaenoptera acutorostrata*	7								
Neobalaenidae	*Caperea marginata*		7							

(1) Blazekovic *et al*.^53^, (2) Cavallero *et al*.^143^, (3) Còlon-Llavina *et al*.^16^, (4) Iñiguez *et al*.^17^, (5) Iñiguez *et al*.^144^, (6) Kleinertz *et al*.^80^, (7) Mattiucci and Nascetti^9^, (8) Mattiucci *et al*.^36^, (9) Shamsi *et al*.^145^, (10) Quiazon *et al*.^146^.

**Table 2:** Global environmental variables used for modelling, provided by GMED^32^.

Variable	Unit	Description
Depth	m	Water depth
Slope	degree	Slope
Land distance	km x 100 (Euclid. Dist.)	Distance (km) to the nearest land cell (water cells only)
Tide average	m	Tides, average of maximum amplitude. These tide model results are from a global 1/4-degree tide model which assimilated tide estimates derived from the TOPEX/Poseidon altimeter.
Wave height	m	Height of waves in scaled discrete classes
Wind speed	m/s	Yearly variations of the surface marine atmosphere over the global oceans (1945–1989)
Surface current	m/s	Monthly average of zonal velocity (UVEL), meridional velocity (VVEL) values in the ocean surface. (2009–2010)
Diffuse attenuation coefficient	1/m	The diffuse attenuation coefficient is an indicator of water clarity. It expresses how deeply visible light in the blue to the green region of the spectrum (490 nm) penetrates into the water column. (2002–2009)
Mean Sea Surface Temperature	°C	Mean sea surface temperature is the temperature of the water at the ocean surface. This parameter indicates the temperature of the topmost meter of the ocean water column. (2002–2009)
Temperature range	°C	Range of sea surface temperature (2002–2009)
Salinity surface	PSS	Salinity indicates the dissolved salt content in the ocean surface (1961–2009)
Salinity bottom	PPT	Long term monitoring of salinity on multiple levels (1871–2008)
Primary production	mgC·m-^2^·/day/cell	Proportion of annual primary production in a cell.
ph	—	Measure of acidity in the ocean surface (1910–2007)

**Table 3:** Modelling parameters.

Species	Occ. records	Env. Variables (abiotic + biotic)	AUC
*Anisakis simplex* s.s.	552	6 + 12	0.952
*A. pegreffii*	332	6 + 8	0.968
*A. berlandi*	78	6 + 5	0.983
*A. typica*	153	6 + 16	0.981
*A. nascetti*	19	6 + 5	0.996
*A. ziphidarum*	105	6 + 4	0.992
*A. brevispiculata*	114	6 + 2	0.978
*A. paggiae*	47	6 + 2	0.961
*A. physeteris*	191	6 + 4	0.972

Number of occurrence records of the *Anisakis* species (Occ. Records); number of environmental variables (abiotic + biotic) used for modelling the habitat suitability of *Anisakis* species and “Area under the Receiver Operator Curve” (AUC) value for the Maxent models.
